# Contour integration in the parafovea and the near periphery: testing the association field account

**DOI:** 10.1098/rspb.2025.1107

**Published:** 2025-09-17

**Authors:** Josephine Reuther, Ramakrishna Chakravarthi, Jasna Martinovic

**Affiliations:** ^1^ School of Psychology, University of Aberdeen, Aberdeen, UK; ^2^ Georg-Elias-Müller Institut für Psychologie, Georg-August-Universität Göttingen, Göttingen, Germany; ^3^ School of Philosophy, Psychology and Language Sciences, The University of Edinburgh, Edinburgh, UK

**Keywords:** contour integration, grouping, peripheral vision, spatial integration, association field

## Abstract

It is essential for object recognition that visual information is appropriately combined. To explain stages of perceptual organization that group elements into contours, the concept of *association fields* has been invoked. Local elements within the boundaries of an association field are grouped to give rise to the perception of a contour if they are appropriately aligned, reasonably similar and close. However, the size of this spatial window remains unclear, as well as how this changes with visual field location. To address this, we studied the combined influence of eccentricity and inter-element spacing on contour detection. Our findings indicate a clear difference in the processing of contours between the parafovea and the periphery. Contour integration in parafoveal regions is efficient and highly stable across a wide range of inter-element spacings and levels of orientation noise. In the periphery, efficient integration is only observed for elements close enough to fall within adjacent receptive fields, while increased inter-element spacings and orientation changes lead to a failure of contour integration. We conclude that two distinct mechanisms underlie contour integration, each with its own spatial extent and tolerance to noise—with the efficient, association field-like mechanism being a characteristic of central vision.

## 1. Introduction

The purpose of visual perception is to enable an organism to successfully navigate environments and interact effectively with objects within them. In humans, this process begins with the extraction of basic features such as orientation, colour and luminance contrast. The representation of such simple features provided by these initial stages is followed by a much more complex and much less understood process of perceptual organization (for a review, see [1]). Perceptual organization is often labelled as mid-level vision to differentiate it from low-level feature processing and high-level semantic processing stages. One of the key goals of mid-level vision is to integrate local elements that belong to the same object while differentiating them from elements that belong to disparate objects. This is a non-trivial process since our visual system has to infer the three-dimensional visual world from two-dimensional images acquired by each eye, and particularly because clutter and occlusion in the real world provide a source of ambiguity as to the contours and boundaries of individual objects. In this context, contour integration is a key putative process that occurs within mid-level vision, linking local elements, such as oriented lines detected by independent feature detectors into a perceived contour. In the early 1990s, in an attempt to develop a model for contour integration, Field *et al.* [2] coined the term *association field*. With a nod to the classical receptive field [3] and its psychophysical analogue the perceptive field [4], the association field refers to the spatial region surrounding a local contour element. Sufficiently associated elements (e.g. similar in appearance and appropriately aligned) within this region are grouped to form a contour, while disassociated elements (e.g. different in colour and spatial frequency structure or misaligned) are segregated from it. Contour integration is thought to be implemented as early as the primary visuocortical area V1 [5–7], although there is some evidence that later regions, such as V3b, initially determine the presence of contours and then recruit earlier regions like V1/V2 for further fine-grained processing [8].

**Figure 1.**
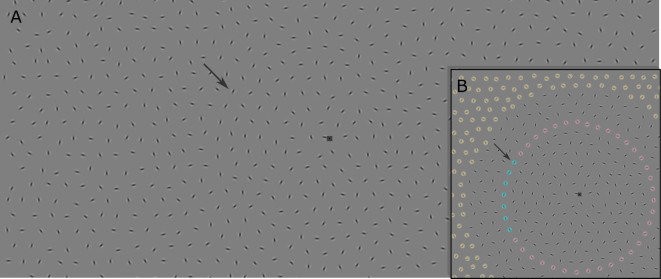
Example stimulus at an intermediate level of eccentricity (10.5°), spacing (1.58°) and wiggle (15°). Arrows indicate the same Gabor patch in the example stimulus (A) and the inset (B) that highlights different parts of the stimulus. Here, the snake consisting of seven Gabor elements (blueish tint) is presented left of fixation. Non-snake elements (reddish tint) extend around the fixation mark to prevent snake detection based on curvature cues alone, and are created in the same way as the snake elements, but with a random orientation. To peripheral Gabors (yellowish tint) 5% positional jitter was added to avoid overly uniform spatial distribution of the far grass elements.

To study contour integration, field, Hess *et al*. developed a now classical contour integration stimulus display formed of spatially evenly distributed oriented bandpass elements (i.e. Gabor patches; see figure 1 for an example), a subset of which is aligned along a path, surrounded by other, randomly oriented background elements (colloquially known as ‘snake in the grass’) [2]. The bandpass nature of the elements is crucial in ensuring that they cannot be linked through simpler and fundamentally different mechanisms based on texture gradients [9,10]. They found that a wide range of factors influences contour integration (see [11] and [5] for a review). Most notable is element orientation [2], where collinearly aligned elements (referred to as a snake — — —) are readily integrated, whereas integration of orthogonal alignment (a ladder | | |) is more effortful and possibly dependent on ocular fixations [2,12,13] (but see [14]) and integration of oblique alignment (a rope */ / /*) is almost at chance [15]. Contour shape also plays an important role. Detectability is best for ‘straight’ snakes and decreases with an increase in curvature [16–18], while maintaining above chance detectability even at high curvature levels [2,19]. Changes in curvature direction (e.g. for an S-shaped snake compared with a C-shaped snake) further reduce detectability [20], particularly when changes are frequent [21] or when element orientation deviates from the local orientation of the underlying path [2]. Taken together, these findings indicate that continuity is paramount for contour integration [5].

Since the association field is defined as a spatial region, one might expect that influences of spatial modulations like inter-element spacing and eccentricity would be well characterized. However, in their original study, while acknowledging that the size of the association field is likely dependent on various stimulus properties, the authors do not provide estimates for the effect of these parameters [2]. For snake stimuli originating from the centre of the visual field, contour integration is still observed for an inter-element spacing of 0.9° visual angle. Beyond that, Field *et al*. [2] propose that association fields would be elongated and comprise an area bigger than a simple receptive field. More recently, contour integration performance in two highly proficient observers was found to decline with an increase in absolute inter-element spacing while still remaining clearly above chance even at an inter-element spacing of 3° visual angle [12]. Beaudot *et al.* [22] report a critical inter-element separation of 4−5° for achromatic stimuli, with 1.2−1.6 times lower values for chromatic stimuli. Using lines instead of Gabor patches, contour integration was also found to be dependent on inter-element spacing, being better for closely spaced (0.9°) than for widely spaced (1.8°) elements [23], with above chance performance even for inter-element separations between 1.6° and 3.6° [24]. While values vary, these findings point to a potential spatial limit for contour integration.

Hess *et al.* [18] proposed that the mechanisms that lead to contour integration may differ between the fovea and the periphery with a putative boundary at roughly 10° eccentricity. For straight paths, contour integration seems well described by linear filtering at fixed orientations regardless of eccentricity. This is in line with the findings that for such paths, contour integration in the periphery barely differs from foveal levels of performance [13,18,25]. However, detection of curved contours in the periphery is strongly impaired as they cannot be processed by linear filters [18]. According to Hess *et al.* [18], the higher tolerance to changes in contour shape in the central visual field is due to inter-cellular linking operations between cells with modest differences in orientation tuning, e.g. through long-range connections. Such linking is assumed to be absent in the periphery, with integration only occurring within fixed orientation bands. Changes in phase, like changes in orientation, require linking across multiple orientation bands. Accordingly, alternating the polarity of Gabor elements was found to impair contour integration specifically in the peripheral visual field [17,18]. But rather than being completely absent beyond 10° eccentricity, contour integration across elements differing in polarity shows a gradual decline with an increase in eccentricity [16]. The decline with eccentricity is even less pronounced for contours of uniform polarity. This implies that the shift from one mechanism to the other might not be strictly sharp.

While the influence of both inter-element spacing and eccentricity has been studied in the past, it is not entirely clear whether and how the two are linked. May & Hess [13] invoked scaling of the association field size with eccentricity as a way to explain the difference in the detectability of snakes and ladders in the periphery. According to this theory, in central vision, association fields are sufficiently small to individuate elements, allowing equally good detection for snakes and ladders. However, in the periphery, where several elements fall into the same association field, ladder elements are likely to fall prey to incidentally occurring alignment with background elements to form spurious snakes, since element associations are stronger for collinear compared with orthogonal alignments. Nevertheless, findings of ‘central’ regions where contour integration remains stable across a range of inter-element spacings (0.1–0.6 times stimulus eccentricity) extending 10° [26] or even 40° into the periphery [25] pose a challenge to the proposal that association field size gradually scales with eccentricity. Yet, in contrast to May & Hess [13], the latter studies presented exclusively collinear and orthogonal Gabor elements in grid structures. Thus, it is unclear whether expanded regions of efficient contour integration are a generalizable observation or specific to highly restrictive stimulus arrangements.

To evaluate the potential co-dependence of contour integration in the periphery on spatial factors, we sought to characterize how eccentricity, inter-element spacing and inter-element orientation change (so-called ‘wiggle’) influence contour detection using a traditional ‘snake in the grass’ stimulus. Presenting snakes with a range of inter-element spacings (0.70°–3.54°) at eccentricities inside (7°), at the boundary (10.5°) and outside (15.75°) of the previously suggested ‘central’ zone of contour integration [18,26], we set out to evaluate the mechanistic properties of the switch from putative efficient long-range linking in the central visual field to less efficient peripheral integration processes in the region between the parafovea and the far periphery. We expected that the proposed properties of the association fields would fit better with the characteristics of the long-range linking process in central vision. In empirical terms, this would be reflected by an interaction of wiggle and spacing with eccentricity, with reduced effects of these two factors for the parafoveal 7.5° eccentricity.

## 2. Methods

### (a) Observers

Twelve observers participated in the experiment (8 female, 4 male and aged 32.3 ± 6.6 years). All but one were experienced observers. Four observers (three authors and a research assistant) were aware of the research question and the types of manipulations used. A simulation-based power analysis confirmed that this sample size was sufficient (see §3). All observers presented with normal or corrected-to-normal vision, assessed on an early treatment diabetic retinopathy study chart [27]. Participants gave written informed consent to participate in the study. All naive observers received monetary reimbursement for their time and effort. The study was approved by the Psychology Ethics Committee at the University of Aberdeen (PEC/4539/2020/9) and is in accordance with the Declaration of Helsinki.

### (b) Apparatus

Stimuli were presented on a linearized 32″ Display ++ screen (CRS, UK) in mono ++ setting, with a refresh rate of 120 Hz and a maximal luminance output of 118.1 cd m^−2^. Temporal dithering algorithms were applied to achieve 16-bit grey values. At the used resolution of 1920 × 1080 pixels, the pixel size was 0.37 mm. The screen was controlled by a Dell Precision T1700 computer with an Nvidia Quadro K420 graphics card. Stimulus presentation was implemented in Matlab (Mathworks, USA) using Psychtoolbox functions [28–30] and the Palamedes toolbox [31]. A 5-key button box (Cedrus RB-530, Cedrus Corporation, USA) was used to record participant responses.

Central fixation was monitored manually using a set-up comprising a LiveTrack Fixation Monitor (CRS, UK) as an infrared light source, a transparent screen mounted on a chin- and forehead-rest to reflect the observer’s eye, and a magnifying infrared camera. The camera streamed the eye reflection to an AD 910A Video Monitor (Sensormatic Electronics Corporation, Boca Raton, FL, USA) at 50 Hz, controlled by a Model 5000 Control Unit (Applied Science Laboratories, Bedford, MA, USA). Trials were rejected online via key release on a standard keyboard.

### (c) Stimuli

Two concentric squares with a side length of 0.1° (black) and 0.5° (dark/light grey), respectively, served as the fixation mark. Gabor elements had a spatial frequency of 2.5 cycles degree^−1^ and a phase-offset (±90°) corresponding to a centre-symmetric profile. The Gaussian window had a standard deviation of 0.16°. Thus, the visible part of the Gabor subtended no more than 0.64°. Simultaneously presented Gabors always had the same randomly assigned polarity. All stimuli of the ‘Snake in the Grass’ display were presented against a mid-grey background (50%, equivalent to 59.05 cd m^−2^) metameric with D65.

Snakes were constructed as described by Mullen *et al.* [32] with minor modifications. Gabor elements were arranged along an imaginary *spine*, each centred on a *vertebra*. Vertebra length determined inter-element spacing, while orientation changes between them defined orientation noise (wiggle). Our snakes consisted of seven elements, with additional vertebrae extending the spine to match the circumference of a circle with a radius equal to the stimulus eccentricity. Wiggle alternated in sign between adjacent vertebrae and varied around the nominal value (±5°). To approximate a circular arrangement, we fitted a regression line to the resulting, relatively straight spine, using the deviations as the distances from the circumference of a perfect circle. Angular correction based on the inter-element spacing was applied to the orientation of the snake elements. ‘Non-snake’ elements along the circle were assigned random orientations, treating them like background noise elements (i.e. grass).

Next, ‘grass’ elements were distributed using iterative Delaney triangulation. Using the *distmesh2d* function [33], we first filled in the area enclosed by the snake and non-snake elements before extending the grass beyond that to fill the entire screen (see figure 1). The distance between the grass elements was based on the average Euclidean distance between snake elements. To avoid overly uniform distribution away from the snake and non-snake elements, positional jitter (5%) was added to all peripheral grass elements that were farther from target eccentricity by more than three times the average distance between the individual snake elements.

The resulting stimulus ensures that all snake elements were presented near the nominal eccentricity, achieving similar detectability and discriminability for all elements. This prevents contrast thresholds from being determined by the farthest Gabor element [26]. Extending non-snake elements into the other half of the display prevents reliance on curvature cues alone. Slight wiggle variability reduces the likelihood of detection through a linear filtering mechanism based on constant curvature (as proposed by [34]), without ambiguity about the stimulus position. Finally, our grass placement method virtually rules out proximity or local density cues (e.g. when snake elements are closer to each other than to surrounding grass elements) that could guide snake detection instead of orientation linking mechanisms.

### (d) Procedure

Contrast thresholds for snake detection were assessed in a spatial two alternative forced choice task for 45 conditions—combining three eccentricities (7°, 10.5° and 15.75°), five inter-element spacings (0.70°, 1.05°, 1.58°, 2.36° and 3.54°; with both eccentricity and spacing in a sequence increasing by a factor of 1.5) and three wiggles (5°, 15° and 25°). The experiment consisted of six 1 h sessions where the participant reported whether the snake stimulus was presented left or right of fixation. Catch-up sessions were added to re-run conditions with poor staircase convergence. Within each session, only one eccentricity was tested. Trials were blocked by spacing and wiggle. The order of sessions was pseudo-random (permuted across participants). Block order was random. Blocks consisted of 80 + 2 trials. The two initial trials (1 per polarity) were not included in the staircase procedure and were added to alleviate the risk of an inattention-related lapse on the first staircase trial. These trials were presented with the same contrast as the starting contrast of the staircases.

Stimulus contrast was controlled using a running-fit procedure. A Weibull-fit (with threshold criterion at 81% correct) was used as the underlying psychometric curve, seeded with a slope of 3.5, a lapse rate of 0.02, with contrast values ranging between 0.001 and 0.95, and an initial threshold estimate of 0.475 (except for eccentricities ≥ 10.5° with spacings ≥ 2.36° and wiggles ≥ 15°, where 0.665 was used). For each condition, the threshold contrast for contour detection was determined by two interleaved staircases, one for positive and one for negative Gabor polarities.

Each trial started with the presentation of the fixation mark in light grey. Button press initiated the trial sequence (central button) and turned the fixation mark dark grey. After 250 ms, the stimulus was presented for a duration of 500 ms. The fixation mark changed back to light grey after another 100 ms to indicate that a response was expected. Participants indicated whether the snake appeared to the left or right of fixation by pressing the respective button box button. Auditory feedback was provided through a beep: high-pitched after incorrect and low-pitched after correct responses. Detection of an eye movement during the trial sequence triggered two shorter, higher pitched beeps and turned the fixation mark pink. Trials with eye movements were discarded and replaced.

Before the main part of each testing session, participants received practice consisting of two blocks of 90 trials. Trials were presented in ascending order of spacing and, within each spacing, increasing wiggle. Targets were positioned at the session’s tested eccentricity. In the first practice block, snakes had higher contrast than the grass (0.75); in the second, both had equal contrast. Practice trials familiarized participants with the stimuli, minimizing differences from top-down strategies or perceptual learning. Catch-up sessions did not include practice but started each block with two high-contrast trials (as in the first practice block) to allow participants to orient attention as blocks could vary in stimulus eccentricity in addition to spacing and wiggle.

### (e) Analysis

To estimate contrast thresholds for each combination of eccentricity, inter-element spacing and orientation wiggle, all trials per condition (80–160; depending on whether the condition was tested again in the catch-up session) were pooled and fed through a running-fit procedure identical to the one that controlled the contrast during stimulus presentation. To determine the reliability of the recomputed thresholds a bootstrapping procedure was used to ascertain a standard error (s.e.) for each of them. Thresholds with a s.e. that exceeded 0.2 were excluded.

The resulting thresholds were submitted to a linear mixed-effects model (LMEM) analysis. To determine the model that best describes the data we started by fitting the most complete, converging model. Next, we removed interactions/factors step by step, when they did not influence the model fit, as indicated by a chi-square difference test. The final model thus encompassed only those factors and their interactions that could not be removed without significantly altering the model fit. The LMEMs were run in R (version 4.2.2) using the *lme4* package [35] and the *nlme* package [36]. Pairwise differences for the estimated means were analysed using the *emmeans* package [37]; the Tukey method was used to correct for multiple comparisons. Power was estimated using the simr package [38].

## 3. Results

**Figure 2.**
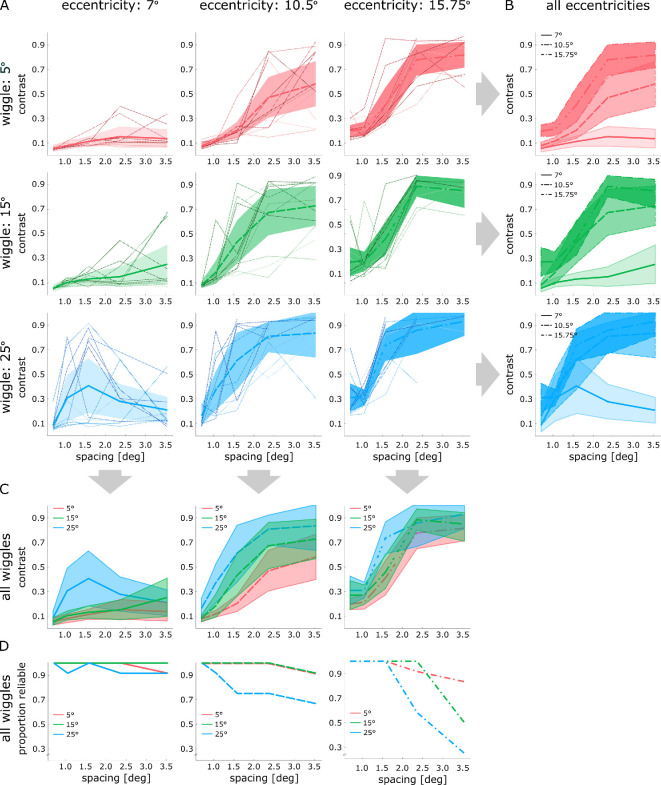
Contrast thresholds as functions of spacing for all eccentricities and wiggles. (A) Individual participant data (thin lines), means (thick lines) and 95% CIs (shading). (B,C) Comparisons of mean thresholds across eccentricities and levels of wiggle, respectively. (D) Proportion of reliably estimated thresholds per spacing for each level of wiggle per eccentricity. Colours indicate the magnitude of orientation wiggle (red: 5°, green: 15° and blue: 25°). Line-types and saturation differentiate between eccentricities (solid: 7°, dashed: 10.5° and dash-dotted: 15.75°; saturation increases with eccentricity).

Figure 2A shows snake localization contrast thresholds for all combinations of stimulus eccentricity and orientation wiggle as functions of inter-element spacing. These data are replotted to facilitate comparison across eccentricities (B), and across the three levels of orientation wiggle (C), respectively.

From visual inspection, it is apparent that the influence of inter-element spacing seems to differ between stimulus presentation in the near periphery and the far periphery. At 7° eccentricity, contrast thresholds seem barely modulated by inter-element spacing (figure 2A,C, column 1), especially when orientation wiggle is low. On the other hand, contrast thresholds increase steeply with an increase in inter-element spacing at stimulus eccentricities of 10.5° and 15.75° (columns 2 and 3). At these eccentricities, contrast thresholds for contour integration reach—and for some participants exceed—the maximum available contrast. Consequently, the proportion of reliable contrast estimates needed for successful contour integration is also influenced by eccentricity, inter-element spacing and wiggle. While the number of unreliable estimates can be partially inferred from the individual participant data in figure 2A (thin lines that end prematurely), figure 2D explicitly shows how the proportion of reliable contrast thresholds varies across all combinations of eccentricity, wiggle and inter-element spacing. It is evident that the proportion of thresholds that failed to converge is low for contours in the parafovea and near periphery, with a slight increase as inter-element spacing and wiggle increase. However, a marked drop in successful threshold convergence is observed for the farther periphery, particularly when the magnitude of wiggle is high.

To statistically evaluate the influence of stimulus eccentricity, inter-element spacing and orientation wiggle, the data were submitted to an LMEM. Inter-participant variability in intercepts was included as a random effect. Models with more complicated random structures (e.g. random slopes) failed to converge. The final model includes all three fixed factors as well as all two-way interactions. The three-way interaction had no influence on the model fit and was thus removed ($\chi^{2}(16)=17.9,p=0.328$). Model selection based on the Akaike Information Criterion yielded the same outcome. Table 1 shows the results of the statistical analysis. An a posteriori sample justification was conducted through simulation from the fitted model, preserving its structure and variance components while reducing fixed effects by 15% to correct for overestimation, as recommended by Kumle *et al.* [39]. This yielded power estimates of 84.20% ± 2.31% for the interaction of eccentricity and wiggle, 95.40% ± 1.35% for the interaction of spacing and wiggle, and effectively 100% for the interaction of eccentricity and wiggle with the lower bound of the confidence interval at 99.63%.

**Table 1.** Best-fitting model.

logit(threshold) ∼ (eccentricity + wiggle + spacing) + ${,}^{2}$1|P			$\Omega^{2}=.772$
	*F*-value (d.f.)	*p*‐value	
eccentricity	255.68 (2, 460)	<0.0001	
wiggle	47.76 (2, 460)	<0.0001	
spacing	166.10 (4, 460)	<0.0001	
ecc:spacing	23.96 (8, 460)	<0.0001	
wiggle:spacing	2.88 (8, 460)	0.004	
ecc:wiggle	3.32 (4, 460)	0.011	

**Figure 3.**
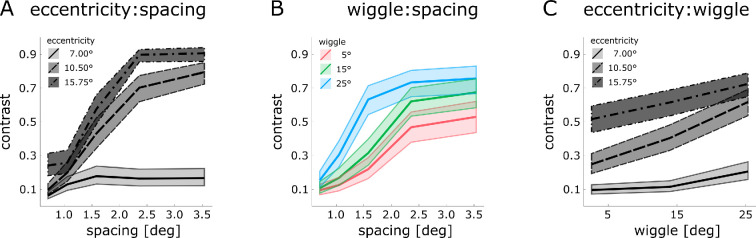
Visualizing the two-way interactions of the best-fitting LMEM. The panels show (A) the interaction between eccentricity and spacing pooled across levels of wiggle, (B) the interaction between wiggle and spacing pooled across all eccentricities and (C) the interaction between wiggle and eccentricity pooled across the five inter-element spacings for our dependent measure (contrast thresholds; estimated means and 95% CI). Line types (indicating stimulus eccentricity: solid: 7°, dashed: 10.5° and dash-dotted: 15.75°) and colours (indicating the level of wiggle: red: 5°, green: 15° and blue: 25°) are used in the accordance with figure 2.

Figure 3 visualizes the two-way interactions from the best-fitting LMEM. The data (estimated means and 95% CI) is averaged across orientation wiggle (figure 3A), eccentricity (figure 3B) or spacing (figure 3C), respectively. To further evaluate the pattern of data observed here, follow-up pairwise comparisons were conducted to assess the effect of spacing per eccentricity (table 2), the effect of spacing per wiggle (table 3) and the effect of wiggle per eccentricity (table 4).

**Table 2.** Pairwise comparisons: effect of spacing at the different eccentricities. Significant differences are indicated in bold. Note that tables 2–4 present the *t*-values and adjusted p-values (Tukey) for each of the pair wise comparisons.

spacing	eccentricity: 7°				eccentricity: 10.5°				eccentricity: 15.75°			
	0.7	1.05	1.58	2.36	0.7	1.05	1.58	2.36	0.7	1.05	1.58	2.36
1.05°	**−3.92** **0.001**				**−4.05** **<0.001**				−0.431 0.993			
1.58°	**−5.81** **<0.0001**	−1.84 0.351			**−9.28** **<0.0001**	**−5.27** **<0.0001**			**−7.08** **<0.0001**	**−6.65** **<0.0001**		
2.36°	**−5.28** **<0.0001**	−1.35 0.663	0.486 0.989		**−14.7** **<0.0001**	**−10.6** **<0.0001**	**−5.28** **<0.0001**		**−15.2** **<0.0001**	**−14.8** **<0.0001**	**−8.51** **<0.0001**	
3.54°	**−5.32** **<0.0001**	−1.42 0.645	0.398 0.995	−0.084 1.00	**−16.5** **<0.0001**	**−12.6** **<0.0001**	**−7.35** **<0.0001**	−2.21 0.176	**−13.4** **<0.0001**	**−13.1** **<0.0001**	**−7.67** **<0.0001**	−0.390 0.995

**Table 3.** Pairwise comparisons: effect of spacing for the different wiggles. Significant differences are indicated in bold.

spacing	wiggle: 5°				wiggle: 15°				wiggle: 25°			
	0.7	1.05	1.58	2.36	0.7	1.05	1.58	2.36	0.7	1.05	1.58	2.36
1.05°	−1.58 0.511				−2.59 0.074				**−4.23** **<0.0001**			
1.58°	**−4.86** **<0.0001**	**−3.28** **0.010**			**−6.59** **<0.0001**	**−4.00** **<0.001**			**−10.7** **<0.0001**	**−6.41** **<0.0001**		
2.36°	**−10.3** **<0.0001**	**−8.77** **<0.0001**	**−5.52** **<0.0001**		**−12.8** **<0.0001**	**−10.2** **<0.0001**	**−6.20** **<0.0001**		**−12.2** **<0.0001**	**−8.14** **<0.0001**	−2.06 0.239	
3.54°	**−11.3** **<0.0001**	**−9.73** **<0.0001**	**−6.55** **<0.0001**	−1.16 0.772	**−13.0** **<0.0001**	**−10.6** **<.0001**	**−6.84** **<.0001**	−1.07 0.824	**−11.7** **<0.0001**	**−7.98** **<0.0001**	−2.40 0.117	−0.490 0.988

**Table 4.** Pairwise comparisons: effect of wiggle at different eccentricities. Significant differences are indicated in bold.

wiggle	eccentricity: 7°		eccentricity: 10.5°		eccentricity: 15.75°	
	5°	15°	5°	15°	5°	15°
15°	−1.27 0.416		**−4.49** **<0.0001**		**−2.43** **0.41**	
25°	**−5.54** **<0.0001**	**−4.31** **0.0001**	**−9.40** **<0.0001**	**−5.14** **<0.0001**	**−5.10** **<0.0001**	**−2.79** **0.015**

Considering the relationship between eccentricity and inter-element spacing (figure 3A and table 2), a clear distinction emerges between stimulus presentation at 7°, compared with 10.5° and 15.75° eccentricity. While contrast thresholds at 7° appear to be stable after an initial increase (between inter-element spacing of 0.7° and 1.05°), contrast thresholds at the higher eccentricities of 10.5° and 15.75° show a clear increase with inter-element spacing. This is confirmed by the results of pairwise *t*-tests at the closest eccentricity, where contrast thresholds were lower for the closest spacing of 0.7° (all *p* <0.001) compared with all other spacings, but did not increase further with an increase in inter-element spacing (all *p* > 0.745). At 10.5°, on the other hand, contrast thresholds increased steadily with an increase in spacing (all *p* <0.001) until levelling off at inter-element spacings of 2.36–3.54° (*p* = 0.950). Likewise, at the farthest eccentricity (15.75°), at intermediate spacing levels of 1.05°–1.58° we observed statistically significant increases in contrast thresholds with an increase in inter-element spacing (all *p* <0.001). However, we did not observe a difference between the closest and the second closest spacing (0.7°–1.05°; *p* = 0.993), and between the farthest and the second farthest spacing (2.36°–3.54°; *p* = 0.993). Note that rather than indicating that stable performance is reached at high-contrast levels, the upper asymptote is likely the result of ceiling effects. This is affirmed by individual participant data (see figure 2D), where the percentage of participants that reach the necessary performance for a contrast threshold to be reliably estimated falls considerably (e.g. below 70% for highest wiggle). That is, especially at the farthest eccentricity and at higher levels of wiggle these thresholds are based on the few observers that were still able to perform the task (but nevertheless required high contrast), while for the remaining observers we were unable to generate sufficient contrast to sustain successful contour integration.

Examining the effect of spacing for the different wiggles (figure 3B and table 3), contrast thresholds appear to increase with wiggle and to show a steeper increase as inter-element spacing gets larger. For low and intermediate amounts of wiggle (5° and 15°), pairwise *t*-tests confirm an increase of contrast thresholds with an increase in spacing for the middle of the tested spacing range (1.05°–2.36°; all *p* <0.013). This was not the case for very close (0.7° and 1.05°, all *p* >0.087) and the very far (2.36° and 3.54°; all *p* > 0.767) spacings. At the highest wiggle, contrast thresholds were also confirmed to increase with an increase in inter-element spacing; however, this was the case from the lowest spacing up to a spacing of 1.58°, after which contrast thresholds did not seem to increase further. This levelling off at higher inter-element distances may again be attributed to ceiling effects where reliable contour integration would have required very high contrasts, surpassing the maximum contrast achievable on our device.

Finally, inspecting the relationship between wiggle and eccentricity (figure 3C and table 4), contrast thresholds were found to increase at all eccentricities with an increase from intermediate to high wiggle (all *p* <0.016). Yet, only at an intermediate eccentricity (10.50°) is a significant increase also found between low and intermediate amounts of wiggle (all *p* <0.0001). Hence, the slope of contrast thresholds as a function of wiggle is consistently steeper at this eccentricity. Overall, in the parafovea (7°) contrast thresholds for contour integration seem to only be influenced by high wiggle, whereas in the periphery an increase in wiggle consistently leads to an increase in the contrast necessary for successful contour integration. That the influence of wiggle does not appear to increase as stimuli move further into the periphery may again be due to contrast thresholds being bound by the maximum contrast levels we could generate on our device.

## 4. Discussion

Association fields have been proposed as the basis for contour integration, a key process that underlies visuo-perceptual organization. Yet, their properties remain poorly understood. In particular, the dependency of contour integration on eccentricity and inter-element spacing has not been fully characterized, leaving a gap in our understanding of long-range spatial vision mechanisms that facilitate the grouping of elements into wholes. Here, we set out to test whether there is a noticeable shift between the outer reaches of the parafovea (at 7° eccentricity) and the periphery (at 10.5° and 15.75° eccentricities), by investigating the influence of inter-element spacing and orientation ‘wiggle’ on contour detection performance. Using ‘snake in the grass’ type stimuli consisting of small Gabor elements, we find that eccentricity, spacing and wiggle interact with each other in a pairwise manner, which indicates that there are at least two separable determinants of the contour integration process.

In the parafovea, we find contour integration to be robust, almost irrespective of changes in inter-element spacing. Element contrast required to perform the task (approx. 17–18%) remains relatively low for the full range of inter-element spacings, even when such spacing was half the stimulus eccentricity. This lack of a (strong) modulation is in line with the scale invariance of contour integration [40]. Furthermore, we observe facilitation at the extremely close range (0.7°), which may be akin to collinear facilitation [41,42] . In contrast, in the periphery, we find contour integration to linearly worsen with inter-element distance with thresholds increasing greatly from close to intermediate inter-element spacing (i.e. 1.05°–2.36°), before completely breaking down for many of our experienced observers (at or above a spacing of 2.36° at 15.75° eccentricity). That is, while contour integration is not limited by inter-element spacing in the central visual field, it is demonstrably so in the periphery (figures 2B and 3A).

The effect of orientation wiggle mirrors the pattern for inter-element spacing (figures 2C and 3C). In the parafovea, only the highest level of wiggle (25°) results in a contrast threshold increase. This is in line with previous studies using high-contrast stimuli that found a decline in contour integration accuracy with an increase in wiggle for *path angles* beyond 15° or 20° [2,12,18]. In the periphery, wiggle has a more marked effect. Contour integration thresholds increase more steeply and for the entire tested range of wiggle (5° to 25°).^1^ Despite this, intriguingly, contrast thresholds remain moderately low (approx. 30%) for very short inter-element spacings (0.7° and 1.05°) even at the farthest eccentricity, which might also be due to collinear facilitation. In summary, while contour integration in the parafovea can tolerate intermediate levels of orientation change, this is generally not the case for the periphery. Considering effects across all eccentricities, wiggle seems to intensify the effect of inter-element spacing, leading to a steeper threshold increase with an increase in inter-element spacing in addition to a slight overall threshold increase.

The aforementioned results, taken together, indicate at least two distinct mechanisms that guide contour integration: a highly efficient mechanism that needs relatively little contrast and is robust to loss of featural or spatial information, operating in the central visual field and a less efficient mechanism in the periphery that is easily susceptible to noise but benefits highly from spatial proximity and featural alignment. Our results thus provide further evidence for a shift in contour integration mechanism between (para)foveal regions and the periphery that had previously been suggested by Hess *et al.* [17] and was recently observed with a more constrained stimulus [26]. The latter study presented horizontal contours arranged in a three-by-three grid stimulus and did not observe contrast thresholds for contour integration to scale with eccentricity. Instead, it was identified that whether integration was successful or not was determined by the eccentricity of the farthest element of the (very short) contour, with stable performance up to about 10° eccentricity. In the present study, keeping the eccentricity of all contour elements at or close to their nominal eccentricity, we again observe performance to be stable for contours presented within parafoveal regions. Thus, the parafovea appears to be highly efficient at integrating contours even at large inter-element spacings and in the presence of orientation noise, and therefore, is likely to rely on long-range linking mechanisms whose efficiency is similar to those in the fovea. In the periphery, however, an efficient mechanism for linking contour shape information, particularly across longer spatial distances (over 1.05°), appears to be absent.

A prominent model of contour integration is the association field model (for a review see [12]). Association field models assume that visual input is structured by linking individual features that fall into a specific region [2]. Early association field models were based on striate cortical cells interconnected by long-range horizontal connections [43]. Although the authors deemed linking across spacings of more than 10° plausible (based on the extent of horizontal connections and receptive field sizes), per the modelled units, connection strength or association strength was assumed to decrease with an increase in inter-element spacing and orientation change. Unfortunately, the model was only tested for the parameters used by Field *et al.* [2] where the maximum separation was 0.9°. Our data challenge the model predictions. First, beyond what we deem a facilitatory effect at the closest inter-element spacing, we do not observe a modulation of contour integration thresholds by inter-element distance in the parafovea. Second, in the periphery, where receptive fields are big, contour integration fails at much closer spacings (approx. 2.36°) than what is deemed plausible.

To understand why contour integration in the periphery is so inefficient, it is necessary to consider its sensitivity to noise. Orientation noise, a deviation of element orientation from the local orientation of the contour, can greatly interfere with contour integration [2]. In the visual periphery, element orientations are represented more noisily and orientation discrimination thresholds are higher [44,45] than in the parafovea. It is conceivable that an increase in noise overlaying the orientation signal in the periphery could account for the difference between the two regions, parafovea and the periphery. However, orientation discrimination thresholds (as one indicator of internal noise) in the periphery are low for the parameters used in our study, particularly in comparison with the added orientation noise that has previously been observed to have a detrimental effect on contour integration (15° [2]). For the element size used in our study, orientation thresholds are approximately 4° at 15° eccentricity and 2–3° at 10° eccentricity [46], whereas the orientation noise we added was a minimum of 5° going up to 25°. Therefore, in line with the conclusions of Hess & Dakin [18], we reject that differences in intrinsic orientation noise across visual field locations drive our results. We also exclude element size as a limiting factor, since as long as element orientation is discriminable, scale invariance does not require cortical magnification [40]. Positional noise, a deviation between element position and the virtual backbone of the contour also increases with eccentricity [47–49]; when added to a centrally presented stimulus, positional noise indeed reduces contour integration performance [18]. Yet again, the levels necessary to introduce a noticeable reduction are high (approx. 10″) and still insufficient to equate foveal and peripheral levels of contour integration [18], making it unlikely as an explanation of the shift between the parafoveal and peripheral outcomes we observe here.

Visual crowding, or the sensitivity to nearby clutter, is another candidate that might account for differences in contour integration between the parafovea and the periphery. Chakravarthi and Pelli [50] argued that crowding and contour integration may be two sides of the same coin. Both arise from the combination of signals across independent feature detectors. Yet, crowding is feature integration gone awry, in that integrated information, stems from multiple distinct objects instead of just one. This leads to an impairment in discriminating object properties, particularly in the periphery, as the region over which information is erroneously combined, referred to as the integration field, increases with eccentricity [51]. May & Hess [13] went even further and based their association field model on assumptions derived from crowding models. First, in keeping with the concept of integration fields used to explain crowding, they assumed that association fields, like integration fields, scale with eccentricity, that association or element linking strength decreases with inter-element distance and that linking is absent beyond the boundaries of the association field. Second, they assumed that linking is alignment-dependent, with stronger linking for collinear than for orthogonal alignment and that it decreases sharply with deviation from either. Last, they assumed that a given element can be linked to at most two others. According to these model assumptions, as the size of the smallest available association field increases, so does the number of nearby elements not belonging to the (target) contour that could potentially be linked to it. As a consequence of the assumed alignment dependence, the probability that such a spurious link to a distractor element is formed increases the more favourable the alignment is (i.e. more collinear).

Applied to snake-in-the-grass type stimuli, the model predicts eccentricity invariance for straight snakes since regardless of how many distractors fall within the smallest available association field, the most favourably aligned element is a neighbouring collinear snake element rather than a neighbouring randomly aligned distractor element. This is exactly what May & Hess [13] observed in their behavioural data, where performance for straight snakes was independent of eccentricity for the tested range (0°–8°). This is also in line with the study discussed earlier [26], where contrast thresholds for the detection of horizontally oriented three-element straight snakes at three different eccentricities (3.5°, 7° and 10.5°) were not limited by inter-element spacing, but by the eccentricity of the farthest element. For straight ladders, the model predicts a sharp decline in contour detection performance with eccentricity since neighbouring distractors are likely to have a more favourable orientation than the neighbouring ladder elements, and thus compete for integration once the smallest available association field includes them. For non-straight snakes, the model predictions should fall somewhere between straight snakes and ladders. That is, the higher the deviation from collinearity (i.e. orientation change between neighbouring snake elements, due to bends in the contour or orientation noise), the higher the likelihood that a link is formed to a more favourably (collinearly) oriented distractor element, thereby possibly reducing (perceived) contour length and hence its detectability.

Predictions for the current study derived from the May & Hess model [13] would strongly depend on the assumed scaling factor for the smallest available association field. Assuming a scaling factor based on Bouma’s rule of thumb, as May & Hess [13] did (i.e. association field radius = 0.5 × eccentricity), and considering only the most proximal elements, which are most likely to be erroneously linked, the association field surrounding a given snake element encompasses one to two and a half times as many grass elements as snake elements. This is the case for all but our most central stimulus (7°) at the widest spacing (3.54°). A more complex model, taking into account the spatial (radial-tangential) anisotropy of the crowding regions would not resolve this. To reconcile the model with the high observed performance in the parafovea and for the closest spacings in the periphery, much smaller association fields would need to be assumed, doubtlessly smaller than those observed for crowding (0.1 × eccentricity at their minimum [52,53]). However, assuming scaling of association field size with eccentricity is problematic in general, as it would predict improved contour integration for inter-element spacings large enough to isolate contour elements from most distractor elements. Instead, in the periphery, we observe contour integration to be good for contours with short inter-element spacings despite relatively high levels of orientation change between snake elements. Yet, for contours with wider spacings thresholds increase before contour integration fails, even when alignment between snake elements is favourable. Taken together, these findings make it unlikely that crowding explains the differences in contour integration in our study, and consequently unlikely that a model proposing a common mechanism for contour integration and crowding is viable.

Contrast sensitivity is a more likely candidate for explaining reduced contour integration in the periphery. After all, Beaudot and Mullen [22] report a narrower critical inter-element distance for chromatic compared with achromatic stimuli, with chromatic contrast sensitivity function being low-pass and lacking the tuning to higher spatial frequencies [54]. Long-range vision has fewer, lower-frequency spatial channels available in the periphery [55]. If putative association field units are a vehicle for accomplishing efficient linkage-based integration that exploits correlations in information across channels, their operation would be severely impeded by the removal of a large subset of finely tuned (high-frequency) channels. While one might consider the possibility that adjusting stimulus size in line with the cortical magnification factor will yield association field like behaviour leading to efficient contour integration in the periphery, it is unlikely to do so since the spatial frequency cut off at 14° eccentricity is 5−6 cycles degree_−1_ [55], which would put a hard constraint on efficient integration. In our study, efficient integration tolerant to orientation changes in the periphery is only evident when two contour elements fall within the same putative perceptive field centre (1° centre and 2−3° total field size at 10−16° eccentricity, according to Ransom–Hogg and Spillman [56], which is consistent with 2.75° estimated at 15.75° eccentricity by [26]). Put together, our findings suggest a breakdown of a long-range linking mechanism in the periphery and a shift towards more effortful processing of noisier outputs from low-level units. Successful contour integration in the periphery seems to occur only under the special circumstance where elements fall within the same perceptive fields. Future studies should measure the bandwidth of contour integration mechanisms in the periphery, using methods similar to those of Dakin & Hess [40] to estimate the characteristics of contrast units that accompany this switch from efficient into effortful contour integration.

## 5. Conclusion

Our study indicates that association fields as a putative mechanism of contour integration may be a specialization of central (i.e. foveal and parafoveal) visual regions. In the periphery, this mechanism breaks down. While crowding may play a role in contour integration, our findings speak against the notion that crowding and grouping/contour integration are two sides of the same coin. Instead, our findings argue for a shift between efficient contour integration in a central region to inefficient integration in the periphery for tasks that require long-range connections that exceed the size of available perceptive fields.

In conclusion, at least two distinct mechanisms are likely to guide contour integration. One is a highly efficient mechanism operating in the central visual field up to an eccentricity of 10°, needs relatively little contrast and is robust to spatial and featural noise. This indicates a long-range, robust and flexible process, in line with the properties of association fields proposed by Field *et al*. [2]. The other is a less efficient mechanism in the visual periphery that is susceptible to featural noise and spatial factors such as element separation and eccentricity.

## Data Availability

Data has been archived on OSF [57].

## Footnotes

^1^
Note that, while performance at 15.75° eccentricity might appear to be less modulated by wiggle comparedwith that at 10.5°, this is likely to be due to the boundary introduced by maximum available contrast (i.e.Michelson contrasts cannot exceed 100%).
